# Colonic EBV-Associated Lymphoproliferative Disorder in a Patient Treated with Rabbit Antithymocyte Globulin for Aplastic Anemia

**DOI:** 10.1155/2012/395801

**Published:** 2012-09-23

**Authors:** Hiroko Sugimoto-Sekiguchi, Haruko Tashiro, Ryosuke Shirasaki, Tomio Arai, Tadashi Yamamoto, Yoko Oka, Nobu Akiyama, Kazuo Kawasugi, Naoki Shirafuji

**Affiliations:** ^1^Department of Hematology/Oncology, Teikyo University School of Medicine, 2-11-1 Kaga, Itabashi-ku, Tokyo 173-8606, Japan; ^2^Division of Pathology, Teikyo University School of Medicine, 2-11-1 Kaga, Itabashi-ku, Tokyo 173-8606, Japan; ^3^Department of Pathology, Tokyo Metropolitan Geriatric Hospital and Institute of Gerontology, 35-2 Sakae-cho, Itabashi-ku, Tokyo 173-0015, Japan

## Abstract

Epstein-Barr-virus- (EBV-) associated lymphoproliferative disorder (LPD) after immunosuppressive therapy for aplastic anemia (AA), in a nontransplant setting, has not been well described. We report one case of colonic EBV-LPD after a single course of immunosuppressive therapy for AA. The patient developed multiple colonic tumors 3 months after receiving immunosuppressive therapy, which consisted of rabbit antithymocyte globulin (ATG), cyclosporine, and methyl-predonisolone. The histological findings of biopsy specimens revealed that atypical lymphocytes had infiltrated colonic glands. Immunohistochemical staining for CD20 was positive, and *in situ* hybridization for EBV-encoded small RNAs was also positive. The EBV viral load in peripheral blood was slightly increased to 140/10^6^ white blood cells. After the cessation of immunosuppressant, the colonic tumors spontaneously regressed, and the EBV viral load decreased to undetectable levels. This is the first report of the single use of rabbit ATG inducing colonic EBV-LPD. Because a single use of immunosuppressive therapy containing rabbit ATG can cause EBV-LPD, we should carefully observe patients receiving rabbit ATG for AA.

## 1. Introduction

Epstein-Barr virus (EBV) is one of the oncogenic viruses, and primary infection usually occurs in childhood. After primary infection, EBV remains latent in the bone marrow and other tissues under immunosurveillance by virus-specific CD8+ cytotoxic T cells and virus-specific CD4+ T cells. EBV reactivation and EBV-associated lymphoproliferative disorder (EBV-LPD) have been increasingly observed in immunodeficient hosts such as patients who received allogeneic hematopoietic stem cell transplantation [1] or solid organ transplantation [2]. EBV-LPD after allogeneic hematopoietic stem cell transplantation (HSCT) is a rare complication; however, it sometimes becomes serious and lethal [3]. The major risk factor for EBV-LPD in a transplant setting is the use of *in vivo* T-cell depletion with antithymocyte globulin (ATG) for the prophylaxis of acute graft versus host disease (GVHD) [1, 4], or a reduced intensity conditioning regimen [5]. In addition, alternative donor stem cell transplantation, that is, an unrelated donor or at least a 2 locus human leukocyte antigen-mismatched-related donor, is associated with an increased risk of EBV-LPD [3]. 

ATG is widely used for the treatment of aplastic anemia (AA) in patients without a suitable donor [6], besides being used for the prevention of acute GVHD in allogeneic HSCT. Whether the use of ATG for AA increases EBV-LPD has not been well established. There are a few reports about EBV-related disease occurring after ATG for AA, in nontransplant setting [7–10]. Here, we report a patient who developed colonic EBV-LPD after immunosuppressive therapy using rabbit ATG, cyclosporine (CSP) and methyl-predonisolone for AA. Furthermore, the lesion disappeared only by the cessation of CSP. 

## 2. Case Presentation

In February 2010, a 55-year-old Japanese man was referred to our hospital due to a bleeding tendency. His blood cell count was hemoglobin (Hb) 10.0 g/dL, platelets 1.5 × 10^4^/*μ*L, white blood cells (WBC) 2.9 × 10^3^/*μ*L with 46% neutrophils and 49% lymphocytes, and absolute reticulocyte number 33,330/*μ*L. Bone marrow examination revealed severe hypocellular marrow without any dysplasia. Chromosomal analysis showed a normal karyotype. A whole-body CT scan showed no abnormal findings. Thus, we diagnosed him with AA (stage 3). Serological studies for human immunodeficiency virus, hepatitis B and C virus, and cytomegalovirus (CMV) were all negative. Anti-EBV antibody titers at diagnosis were viral capsid antigen (VCA) IgG-positive, VCA IgM-negative, and EBV nuclear antigen- (EBNA-) positive. The quantities of EBV-DNA viral load in peripheral blood using the RT-PCR method were not measured at diagnosis. He depended on regulatory red blood cell and platelet transfusions. 

**Figure 1:**
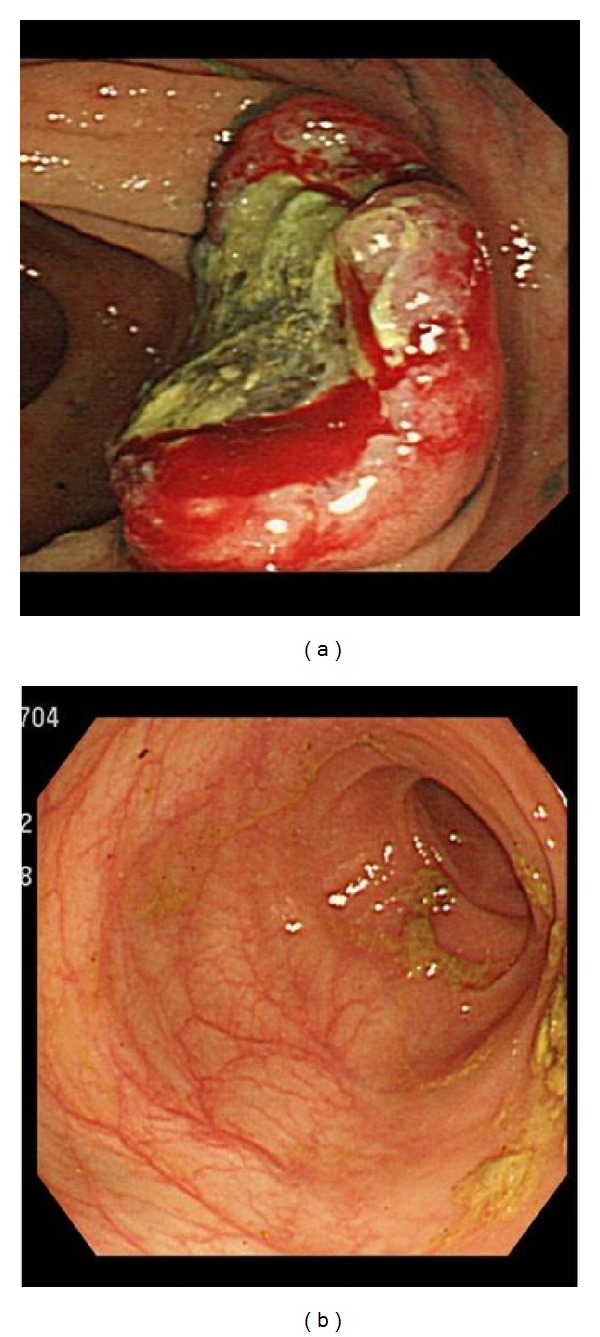
(a) Colonoscopic findings at diagnosis. Multiple tumors at the ascending colon are observed. (b) Colonoscopic findings one month after the cessation of CSP. No tumors are observed.

**Figure 2:**
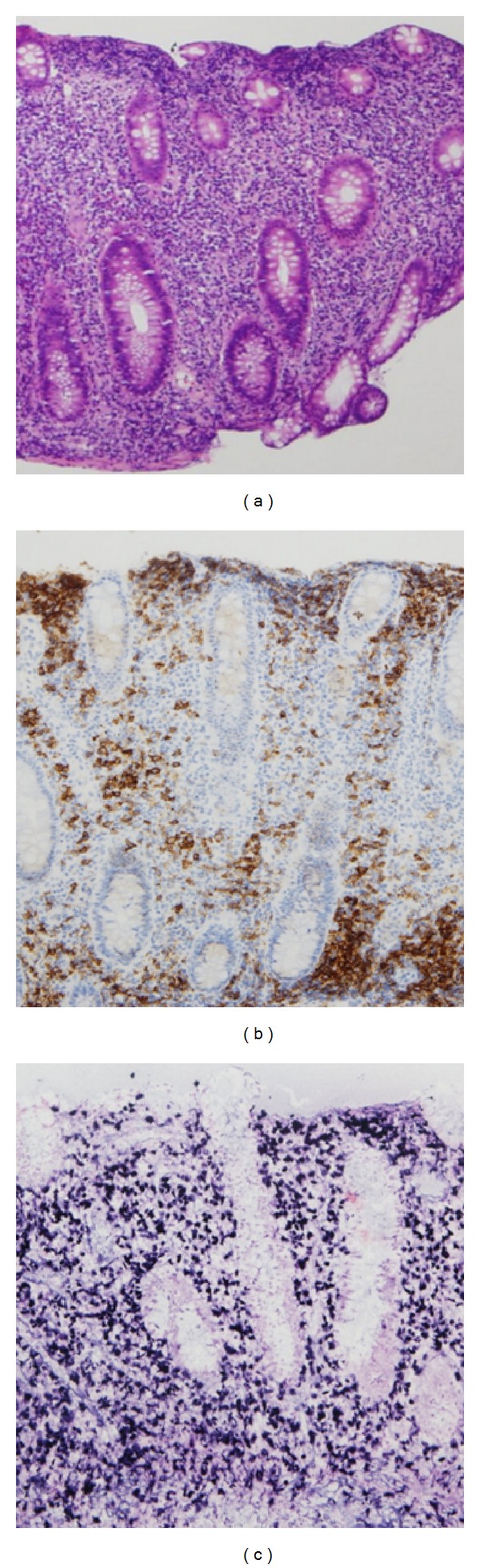
Histologic findings of biopsy specimens. (a) Atypical lymphocytes, lymphocytes, and neutrophils infiltrate the colonic lamina propria. Mildly atrophied crypts with goblet cell depletion are also observed (H&E, original magnification ×10). (b) Immunohistochemical staining with anti-CD20 antibody shows a diffuse reaction (original magnification ×25). (c) *In situ* hybridization for EBER reveals positive staining (original magnification ×25).

Because he had no related HLA-identical donor, he was treated with immunosuppressive therapy consisted of methyl-predonisolone (125 mg/day days 1–5, then tapered), CSP (6 mg/kg/day, orally), and rabbit ATG (3.75 mg/kg/day, days 1–5) in May 2010. The dose of rabbit ATG was decided according to the guideline for the diagnosis and management of AA of the British Society of Haematology [11]. On day +9 after immunosuppressive therapy, his lymphocyte count decreased to 0/*μ*L. Although, after day +13, his lymphocyte count increased gradually, it was maintained on the level below 100/*μ*L. He developed no documented infection, and he was discharged with a regulatory blood transfusion dependency. In August 2010, he developed severe anemia and needed frequent red blood cell transfusions. Moreover, the serum LDH gradually increased to 358 IU/L from a normal range (119–229 IU/L). He developed general malaise and appetite loss. He was afebrile and did not develop superficial lymphadenopathy. Because occult blood tests in stools were positive, he underwent a colonoscopy, which revealed multiple tumors of the ascending, transverse, and descending colons (Figure 1(a)). All biopsy specimens from colonic tumors showed the proliferation of atypical lymphocytes (Figure 2(a)). Immunostaining revealed that the atypical lymphocytes were CD20+ (Figure 2(b)), CD79a+, CD3−, and CD5−. Immunocytochemistry studies for EBV encoded small RNAs (EBER) involving *in situ* hybridization were positive (Figure 2(c)). The PET/CT showed no lymphadenopathy other than the colonic tumors. On day +84 after immunosuppressive therapy, the EBV viral load was slightly increased to 140/10^6^ WBC. He was diagnosed with EBV-LPD. He received only CSP as immunosuppressive therapy at day +84. The serum concentration of CSP was 333 ng/mL on day +90. We stopped the administration of CSP on day +97. After cessation, his symptoms gradually improved, and the serum LDH level decreased to the normal range. His lymphocyte counts gradually increased to 300/*μ*L. One month after the cessation of CSP, he underwent colonoscopy again, which showed regression of the tumors (Figure 1(b)). Two months after the cessation of CSP, the EBV viral load was below the detectable level. In February 2011, he had achieved a partial hematological response and was independent of blood transfusion. At this moment, 2 years later, no EBV-LPD occurred. 

## 3. Discussion

EBV-LPD in patients undergoing allogeneic HSCT is increasing, because pre- and posttransplantation settings have diversified. The risk factors associated with the development of EBV-LPD after allogeneic HSCT have yet to be a higher degree of immunosuppression, including ATG. However, it is not determined whether the therapeutic use of ATG for AA, in a nontransplant setting, is a risk factor for EBV-LPD.

There are a few reports concerning EBV-related [7–10] or EBV-nonrelated [12, 13] LPD after immunosuppressive therapy for AA. A brief summary of EBV-LPD in a nontransplant setting is shown in Table 1. Wondergem et al. [10] reported EBV-associated diffuse large B-cell lymphoma in a patient with severe AA who was treated with rabbit ATG as a second course of immunosuppression. They suggested the feasibility of monitoring EBV reactivation in patients being treated with rabbit ATG as a second course of immunosuppression. In addition, Calistri et al. [7] reported the case of a patient who developed infectious mononucleosis, after immunosuppressive therapy with CSP and two courses of ATG (first course was rabbit ATG, and second was equine) for AA. These two cases were administered both rabbit and equine ATG. The second course of ATG may be a risk factor for EBV-LPD after immunosuppressive therapy for AA. Rabbit ATG is considered to be more immunosuppressive than equine ATG. In a study comparing rabbit with equine ATG, lymphocytopenia was more prolonged and the median peak EBV copy number was higher in the rabbit ATG than in the equine ATG group [14, 15].

Here, we report a patient who developed colonic EBV-LPD after rabbit ATG as the first course of immunosuppressive therapy consisted of rabbit ATG, CSP, and methyl predonisolone for AA. Due to the fact that we did not perform colonoscopy before immunosuppressive therapy, there is a possibility that his tumors existed before immunosuppressive therapy. However, his colonic tumors were diminished, and the EBV viral load was decreased only by the discontinuation of CSP. These findings suggested that his EBV-LPD may have occurred due to the suppression of his cellular immunity. To our knowledge, this is the first report of EBV-LPD caused by one course of rabbit ATG for AA, and, moreover, that it could be diminished only by the cessation of CSP. In contrast to other reported cases, in our patient, the EBV viral load was relatively low and his clinical symptoms were mild. This may reflect the lighter degree of immunosuppression by single course of ATG. On the other hand, the optimal given dose of ATG has not become settled yet. Although we administered 3.75 mg/kg of rabbit ATG for 5 days according to the British guideline, there are several reports of lower doses of rabbit ATG for immunosuppressive therapy [16–18]. The dose of 3.75 mg/kg for 5 days may have been too strong for immune suppression. Further investigation is needed to explore the optimal doses of rabbit ATG for AA. 

EBV-LPD in allogeneic HSCT is sometimes lethal [19] and is needed for early intervention. To treat early frequent monitoring of the EBV-DNA load by quantitative real-time PCR has been suggested [2]. However, Wakabayashi et al. [20] reported that weekly monitoring for EBV-DNA could not prevent the development of EBV-LPD after allogeneic bone marrow transplantation for severe AA. Furthermore, in nontransplant setting, Scheinberg et al. [14] reported their findings on CMV and EBV reactivation following immunosuppressive therapy for AA, in which they state that although EBV reactivation was observed, in the majority of patients no EBV-related disease occurred. Thus, the authors concluded that routine monitoring of the viral load is not necessary for AA patients who have received immunosuppressive therapy. However, as mentioned above, EBV-LPD, including our case, was demonstrated also after immunosuppressive therapy for AA. It remains controversial whether routine monitoring of the EBV-DNA viral load is necessary or not.

Although, in our case, the EBV-LPD disappeared only by the cessation of CSP, EBV-LPD cannot usually be effectively controlled by the dose reduction or discontinuation of immunosuppressants alone, in an allogeneic HSCT setting. And EBV-specific cytotoxic T cells with donor lymphocyte infusion and rituximab are available as a therapeutic option [21]. Furthermore, adoptive immunotherapy with EBV-specific T cells has been investigated for EBV-LPD in transplant settings [2]. 

In conclusion, EBV-related disease may occur after a single course of rabbit ATG for AA. We should observe patients carefully not only during but also after immunosuppressive therapy for AA. Once EBV-LPD occurs, cessation of the immunosuppressant may be an effective treatment option in a nontransplant setting. 

## Figures and Tables

**Table 1:** Summary of EBV-LPD after ATG for aplastic anemia in a nontransplant setting.

Age, gender	Type of ATG	Dose of ATG	Other drugs	Months to diagnosis from ATG	EBV viral load	Type of LPD	Previous treatment for AA	Treatment for LPD	Outcome	Reference
38, M	Equine	3.5 mg/kg NR	mPSL, CSP	1>	30,000/150,000 cells	IM	rATG	Rit	CR	[7]
36, M	Equine	0.75 mL/kg 8 days	CSP	1>	NR	EBV-LPD	NR	NR	Dead	[8]
55, M	Equine	NR	None	1	60,060/mL	EBV-LPD	ATG*	GCV**, FCV** Rit	NR	[9]
42, F	Rabbit	12.5 mg/kg 4 days	CSP	1>	4 × 10^6^/mL	DLBCL	eATG	Rit, CPA	CR	[10]
55, M	Rabbit	3.75 mg/kg 5 days	mPSL, CSP	3	140/10^6^ WBC	EBV-LPD	None	Cessation of CSP	CR	Our case

NR: not reported, IM: infectious monocytosis, rATG: rabbit ATG, eATG: equine ATG, Rit: rituximab, CR: complete remission, EBV-LPD: Epstein-Barr-virus-associated lymphoproliferative disorder, GCV: ganciclovir, FCV: foscarnet, DLBCL: diffuse large B-cell lymphoma, CPA: cyclophosphamide, and CSP: cyclosporine.

*This patient received autologous peripheral blood stem cell transplantation for Burkitt lymphoma before development of aplastic anemia.

**For concurrence of cytomegalovirus reactivation.

## References

[1] Van Esser JWJ, Van Der Holt B, Meijer E (2001). Epstein-Barr virus (EBV) reactivation is a frequent event after allogeneic stem cell transplantation (SCT) and quantitatively predicts EBV-lymphoproliferative disease following T-cell-depleted SCT. *Blood*.

[2] Shaffer DR, Rooney CM, Gottschalk S (2010). Immunotherapeutic options for Epstein-Barr virus-associated lymphoproliferative disease following transplantation. *Immunotherapy*.

[3] Meijer E, Cornelissen JJ (2008). Epstein-Barr virus-associated lymphoproliferative disease after allogeneic haematopoietic stem cell transplantation: molecular monitoring and early treatment of high-risk patients. *Current Opinion in Hematology*.

[4] Bacigalupo A (2005). Antilymphocyte/thymocyte globulin for graft versus host disease prophylaxis: efficacy and side effects. *Bone Marrow Transplantation*.

[5] Cohen J, Gandhi M, Naik P (2005). Increased incidence of EBV-related disease following paediatric stem cell transplantation with reduced-intensity conditioning. *British Journal of Haematology*.

[6] Rosenfeld S, Follmann D, Nunez O, Young NS (2003). Antithymocyte globulin and cyclosporine for severe aplastic anemia: association between hematologic response and long-term outcome. *Journal of the American Medical Association*.

[7] Calistri E, Tiribelli M, Battista M (2006). Epstein-Barr virus reactivation in a patient treated with anti-thymocyte globulin for severe aplastic anemia. *American Journal of Hematology*.

[8] Raghavachar A, Ganser A, Freund M, Heimpel H, Herrmann F, Schrezenmeier H (1996). Long-term interleukin-3 and intensive immunosuppression in the treatment of aplastic anemia. *Cytokines and Molecular Therapy*.

[9] Viola GM, Zu Y, Baker KR, Aslam S (2010). Epstein-Barr virus-related lymphoproliferative disorder induced by equine anti-thymocyte globulin therapy. *Medical Oncology*.

[10] Wondergem MJ, Stevens SJC, Janssen JJWM (2008). Monitoring of EBV reactivation is justified in patients with aplastic anemia treated with rabbit ATG as a second course of immunosuppression. *Blood*.

[11] Marsh JCW, Ball SE, Cavenagh J (2009). Guidelines for the diagnosis and management of aplastic anaemia. *British Journal of Haematology*.

[12] Dorr V, Doolittle G, Woodroof J (1996). First report of a B cell lymphoproliterative disorder arising in a patient treated with immune suppressants for severe aplastic anemia. *American Journal of Hematology*.

[13] Suzuki Y, Niitsu N, Hayama M (2009). Lymphoproliferative disorders after immunosuppressive therapy for aplastic anemia: a case report and literature review. *Acta Haematologica*.

[14] Scheinberg P, Fischer SH, Li L (2007). Distinct EBV and CMV reactivation patterns following antibody-based immunosuppressive regimens in patients with severe aplastic anemia. *Blood*.

[15] Scheinberg P, Nunez O, Weinstein B (2011). Horse versus rabbit antithymocyte globulin in acquired aplastic anemia. *New England Journal of Medicine*.

[16] Afable MG, Shaik M, Sugimoto Y (2011). Efficacy of rabbit anti-thymocyte globulin in severe aplastic anemia. *Haematologica*.

[17] Di Bona E, Rodeghiero F, Bruno B (1999). Rabbit antithymocyte globulin (r-ATG) plus cyclosporine and granulocyte colony stimulating factor is an effective treatment for aplastic anaemia patients unresponsive to a first course of intensive immunosuppressive therapy. Gruppo Italiano Trapianto di Midollo Osseo (GITMO). *British Journal of Haematology*.

[18] Garg R, Faderl S, Garcia-Manero G (2009). Phase II study of rabbit anti-thymocyte globulin, cyclosporine and granulocyte colony-stimulating factor in patients with aplastic anemia and myelodysplastic syndrome. *Leukemia*.

[19] Peres E, Savasan S, Klein J, Abidi M, Dansey R, Abella E (2005). High fatality rate of Epstein-Barr virus-associated lymphoproliferative disorder occurring after bone marrow transplantation with rabbit antithymocyte globulin conditioning regimens. *Journal of Clinical Microbiology*.

[20] Wakabayashi S, Ohashi K, Hanajiri R (2010). Rapidly progressive Epstein-Barr virus-associated lymphoproliferative disorder unpredictable by weekly viral load monitoring. *Internal Medicine*.

[21] McGuirk JP, Seropian S, Howe G, Smith B, Stoddart L, Cooper DL (1999). Use of rituximab and irradiated donor-derived lymphocytes to control Epstein-Barr virus-associated lymphoproliferation in patients undergoing related haplo-identical stem cell transplantation. *Bone Marrow Transplantation*.

